# Subtle Gardeners: Inland Predators Enrich Local Topsoils and Enhance Plant Growth

**DOI:** 10.1371/journal.pone.0138273

**Published:** 2015-09-18

**Authors:** José M. Fedriani, Pedro José Garrote, María del Mar Delgado, Vincenzo Penteriani

**Affiliations:** 1 Centre for Applied Ecology "Prof. Baeta Neves"/InBIO, Institute Superior of Agronomy, University of Lisbon, Tapada da Ajuda, Lisboa, Portugal; 2 Department of Conservation Biology, Estación Biológica de Doñana, C.S.I.C., c/ Americo Vespucio s/n, Seville, Spain; 3 Metapopulation Research Group, Department of Biosciences, University of Helsinki, Helsinki, Finland; 4 Research Unit of Biodiversity (UMIB, UO-CSIC-PA), Oviedo University - Campus Mieres, Mieres, Spain; Estacion Experimental de Zonas Áridas (CSIC), SPAIN

## Abstract

Inland vertebrate predators could enrich of nutrients the local top soils in the area surrounding their nests and dens by depositing faeces, urine, and prey remains and, thus, alter the dynamics of plant populations. Surprisingly, and in contrast with convincing evidence from coastal habitats, whether and how this phenomenon occurs in inland habitats is largely uncertain even though these habitats represent a major fraction of the earth's surface. We investigated during two consecutive breeding seasons the potential enrichment of the top-soils associated with inland ground-nesting eagle owls *Bubo bubo*, as well as its possible consequences in the growth of two common annual grasses in southern Spain. Top-soils associated with owl nests differed strongly and significantly from control top-soils in chemical parameters, mainly fertility-related properties. Specifically, levels of available phosphorus, total nitrogen, organic matter, and available potassium were 49.1, 5.6, 3.1, and 2.7 times higher, respectively, in top-soils associated with owl nests as compared to control top-soils. Germination experiments in chambers indicated that nutrient enrichment by nesting owls enhanced seedling growth in both annual grasses (*Phalaris canariensis* and *Avena sativa*), with seedling size being 1.4–1.3 times higher in owl nest top-soils than in control top-soils. Our experimental study revealed that pervasive inland, predatory birds can profoundly enrich the topsoil around their nests and, thus, potentially enhance local vegetation growth. Because diverse inland vertebrate predators are widespread in most habitats they have a strong potential to enhance spatial heterogeneity, impinge on plant communities, and exert an overlooked effect on primary productivity worldwide.

## Introduction

Predators are well-known for their indirect positive effect on many plant populations and communities [1, 2]. These indirect effects often involve the numerically control of primary consumer populations as well as the alteration of herbivore foraging in response to perceived predation risk [3]. Also, some vertebrate predators enhance plant dynamic and expansion by dispersing seeds, either directly [4] or indirectly [5]. In some coastal habitats, seabirds are known to deposit large amounts of guano and prey carcasses near their nests [6–9]. This generates spatial variation in biotic and abiotic topsoil properties at a microscale that often provide unique regeneration niches for many plant populations and communities and, as a result, promote habitat productivity and diversity [10–12]. Surprisingly, despite the large spatial extension and ecological importance of inland habitats, very little is known about the potential of inland predators (i.e. those typically occupying habitats away from the coast) to enrich soils and the presumed coupled effects on plant populations and communities. Detailed investigations concerning the potential topsoil nutrient enrichment by inland predator activity and its ecological outcomes on plant communities are thus clearly needed.

Many inland vertebrate predators, and especially central-place foragers (e.g. birds, some mammals), enrich the top soil in the area surrounding their nests and dens by depositing faeces, urine, and prey remains [4, 13–16]. Inland predators such as diurnal and nocturnal raptors, bee-eaters, larks, wheatears, nightjars, and tetraonids place their nests on the ground and thus are likely to generate local topsoil nutrient enrichment. Whereas the most immediate effects of those widespread predators is predictable (i.e. nutrient enrichment), whether and how inland predators locally alter the growth and dynamic of plant populations is a intriguing overlooked question with potential repercussions for a large part of terrestrial habitats.

This experimental study illustrates how inland predators enrich the topsoil in the vicinity of their nests, as well as its coupled effects on seedling growth and emergence. To this end, we chose to study a population of ground nesting eagle owls *Bubo bubo* L. in south-western Spain. These ground-nesting predators are known to deposit faeces and amass prey remains in the immediate surroundings of their nests [17]. Preliminary field observations suggest that owls appear to enrich the topsoil associated to their nests and also that a high concentration of vegetation cover tends to occur within owl nest vicinities. To investigate the potential effect of soil enrichment on plant growth, we used two common annual grasses widely distributed and frequently used in experimental germination trials, the Canary grass *Phalaris canariensis* L. and the common oat *Avena sativa* L. [18–21]. Because both species are well adapted to poor habitats but *A*. *sativa* reach sizes several times that of *P*. *canariensis*, we expected that, for a given seedling number, the effect of topsoil enrichment on seedling growth would be more marked in the larger *A*. *sativa* seedlings, as they are likely to have higher nutrient requirements than *P*. *canariensis*. Specifically, our experimental approach enables us to address the following four questions: (1) Do owls alter chemical and/or physical topsoil properties of the top-soil surrounding their nests? (2) Is there any difference in annual seedling growth between owl nests top-soils and control top-soils? and, if so, (3) is such difference consistent between annual species? If both top-soil properties and seedling growth are indeed altered by nesting owls, (4) to what extent and how are those owl-induced changes linked?

## Methods

### Study area

The study was carried out during the owl breeding seasons (February-June) of 2013 and 2014 in the Sierra Norte of Seville (37°30'N, 06°03'W, SW Spain). The area comprises an artificial lake (called "Embalse del Gergal") at the end of the Rivera de Huelva. The area of the lake is approximately 250 ha and it is surrounded by small low-level hills (60–200 m in altitude). These hills are characterised by a typical Mediterranean scrubland with an understory of mastic tree *Pistacea lentiscus* L., crimson-spot rock rose *Cistus ladanifer L*., Portuguese heath *Erica lusitanica Rudolph*, broom *Sarothamnus scoparius* L., and *Asparragus* spp. The overstory comprises holm oak *Quercus ilex ballota* L., cork oak *Quercus suber* L., gall oak *Quercus faginea* Lam., pine trees *Pinus pinea L*., olive trees *Olea europea* L., and eycalyptus *Eucalyptus sideroxylon* A. Cunn. The local community of herbs and annual grasses is very diverse [18], including several common ruderal and cultivated species such as five *Avena* spp. (e.g. *Avena barbata* Pott, *A*. *sativa*, *Avena sterilis* L.) and six *Phalaris* spp. (*Phalaris coerulescens* Desf., *P*. *canariensis*, *Phalaris minor* Retz. [18]).

The area has a typical Mediterranean climate with an annual average temperature of 18.5°C. It is characterized by hot summers with average maximum monthly temperatures above 30°C and temperate winters with average minimum monthly temperatures around 7.5°C. Average annual rainfall is around 600 mm. Maximum rainfall occurs in winter whereas in summer rain is practically absent. Rainfall is characterized by high irregularity both intra-annually and inter-annually with severe droughts [22].

The eagle owl is the largest European owl, which mostly preys on rabbits (*Oryctolagus cuniculus* L.) and rats (*Rattus* spp.) in the study area [23] and their remains are often found in the owl nest vicinity. Such prey remains, as well as owl faeces, pellets, and feathers eventually decompose, probably having an effect on top-soil composition. Within our study area, all eagle owl nests are located on ground with a mean (±1SD) number of fledglings of 2.18±1.03 per brood (range: 1–4 chicks; [24]).

### Effect of eagle owl nests on top-soil chemical and physical properties

To assess the effect of owl nests on the adjacent top-soil, we collected top-soil samples by extracting the superficial ~15 cm top-soil layer (~300g) at each nest (n = 21). Furthermore, for each focal nest, we collected a similar top-soil sample from a comparable microsite (e.g. beneath a shrub *vs*. an open microsite) which was located ~15 m away from the nests (i.e. control samples). By similar microhabitat for each pair of (control and nest) soil samples, we controlled for any potential effect of eagle owl microhabitat nest selection on top soil proprieties. All top-soil samples were sieved (<2mm) to separate gravel and non-soil components. Top-soil analyses were conducted at the Instituto de Recursos Naturales y Agrobiología (CSIC, Seville, Spain) following standard top-soil analytical procedures [25,26]: texture (% sand, silt, gravel and clay; Bouyoucos’s densimeter), pH (extract in 1:2.5 water to KCl), total carbonate content (% CaCO_3_; manometric method), organic matter (%; Walkley and Black method), available phosphorus (mg/kg; Olsen method), available potassium (mg/kg; acetate extraction), and total nitrogen (%; Kjeldahl method).

### Effect of owl nests on seedling growth

As we found that nests had indeed a nutrient enrichment effect on adjacent top-soils (see Results), we asked whether such enrichment could have consequences for seedling growth. To assess this prospect, we utilized the same top-soil samples employed for the analysis of the chemical and physical properties to conduct a germination experiment using the abovementioned Canary grass and common oat. Both species show generally high germination rates [19–21] and, thus, are appropriate for experimentally evaluating the effect of top-soils on seedling growth.

In 2013 and 2014 we performed germination experiments considering 21 and 8 nests, respectively (Table 1). Seeds were sown in separate pots (8x8x18 cm). In each pot, we sowed 6 seeds of one species about 2–3 mm deep. To control for potential confounding factors, the pots were placed in a chamber with controlled temperature (25–30°C), humidity and photoperiod (12/12), and they were watered two times a week until seedlings completed their growth (~30–40 cm in length). Date of emergency and length were recorded once per week. When seedlings completed their growth, we removed them from the pots, measured their length and diameter using ruler and digital caliper (at the nearest 1.0 and 0.05 mm, respectively). The dry weight of each seedling was also measured after three days in an oven at 50°C [27]. In 2013 we sowed 504 seeds (21 nests x 2 plant species x 2 top-soil types x 6 seeds per pot) and in 2014 we sowed 192 seeds (8 nests x 2 plant species x 2 top-soil types x 6 seeds per pot).

**Table 1:** Variation in chemical and physical characteristics of soil samples considered in this study and corresponding univariate tests using GLM procedure for differences between topsoils of owl nests *vs*. control sites.

	Range	Mean	Coefficient of variation (%)	R^2^	*F*_*1*,*40*_	*P*
**Sand**	31.2–90.3	63.42	19.50	0.008	0.32	0.575
**Silt**	5.4–43.5	23.65	34.74	0.00	0.00	0.960
**Clay**	3.2–32.4	12.91	47.33	0.035	1.47	0.233
**pH**	4.48–8.42	5.83	13.06	0.085	3.72	0.061
**Organic matter**	0.12–23.5	8.28	72.96	0.498	39.62	**<.0001**
**Phosphorus**	1.7–545	120.08	119.59	0.660	77.72	**<.0001**
**Potassium**	45–2490	383.17	113.80	0.175	8.22	**0.007**
**Nitrogen**	0.04–2.04	0.617	90.31	0.375	62.59	**<.0001**

Field work was done under the framework of the following authorizations: the Junta de Andalucia–Consejería de Medio Ambiente permit nos. SCFFSAFR/ GGG RS-260 / 02 and SCFFS-AFR/CMM RS-1904 / 02.

### Statistical analyses

Multivariate analysis of variance (MANOVA) in GLM procedure of SAS [28] were done to test overall differences in chemical and physical traits between top-soils from owl nests and from controls. Once overall significant differences were detected, we applied univariate analyses for each soil variable. Because sets of top-soil variables are often closely correlated [29–32], and this multicollinearity may affect the reliability of significance tests for individual effects, scores of top-soil samples on rotated principal components were also used as top-soil descriptors [32]. The principal components analysis was carried out on the correlation matrix using SAS procedure FACTOR. To estimate the principal components, the original variables were standardized to unit variance. The three principal components with associated eigenvalues >1 were retained. After the components were estimated, their interpretation is most straightforward by rotating them. In the rotated pattern matrix all the coefficients are close to 0 or ±1 and, thus, it is easier to interpret than a pattern with many intermediate elements [28].

Data on percentages of *P*. *canariensis* and *A*. *sativa* seedling emergence and seedling size (length, diameter, and dry weight) were analyzed fitting generalized linear mixed models using Proc Glimmix in SAS [33]. The effects of plant species and top-soil (i.e. from owl nest *vs*. control) as well as their second order interaction were specified in the models as fixed effects, whereas each pair of (control and nest) soil samples (i.e. experimental block) and year (2013, 2014) were included as random factors. By specifying block as a random factor, we statistically accounted for the paired nature of our sampling protocol. A significant interaction between top-soil and plant species would indicate spatial inconsistency across species in the effect of the top-soil. For proportions (e.g. seedling emergence) and size response variables, we specified in the models the appropriate error (Binomial and Normal, respectively) and the canonical link functions [33]. To compare the effects of different levels of any significant main factor, we calculated the difference between their least-square means. When the interaction between any two factors was significant, we performed tests for the effect of a given factor at the different levels of the other factor (“tests of simple main effects”), using the SLICE option in the LSMEANS statement of the MIXED procedure [33].

The effects of top-soil type and plant species and their interaction on the speed of seedling emergence were tested using failure-time analyses, by fitting Cox proportional hazard regression models using the S-Plus function coxph [34]. Data consisted of the number of days between sowing and seedling emergence. Because all seeds considered emerged seedlings by the end of the study, we used uncensored data [27]. Experimental block was included in the model as a “frailty” or random term, and the significance of each factor and interaction was evaluated by backwards-stepwise elimination from the full model [34]. In comparing successive models, we calculated the double absolute difference of their respective expectation-maximization (EM) likelihood algorithms, and compared that value against a chi-square with k-1 degrees of freedom, k being the number of levels (or combination of levels) of the factor (or interaction) being tested. For the frailty factor we also assumed a chi-square distribution with one degree of freedom.

## Results

### Variation in top-soil properties

All measured top-soil parameters exhibited considerable variability when all the top-soil samples were considered together (Table 1). Top-soil types differed significantly in mean top-soil chemical parameters, both for each parameter considered individually (GLM univariate tests; Table 1) and when all parameters were treated simultaneously in a multivariate analysis of variance (MANOVA, F_8, 33_ = 13.59, *P*<0.0001). Broad ranges occurred mostly for fertility-related attributes such as organic matter (0.12–23.50%), total nitrogen (0.04–2.04%), available phosphorus (1.70–545.0 mg/kg) and available potassium (45–2490 mg/kg). However, there were no differences between top-soil types in texture-related parameters (all *P*-values > 0.233, Table 1).

**Fig 1:**
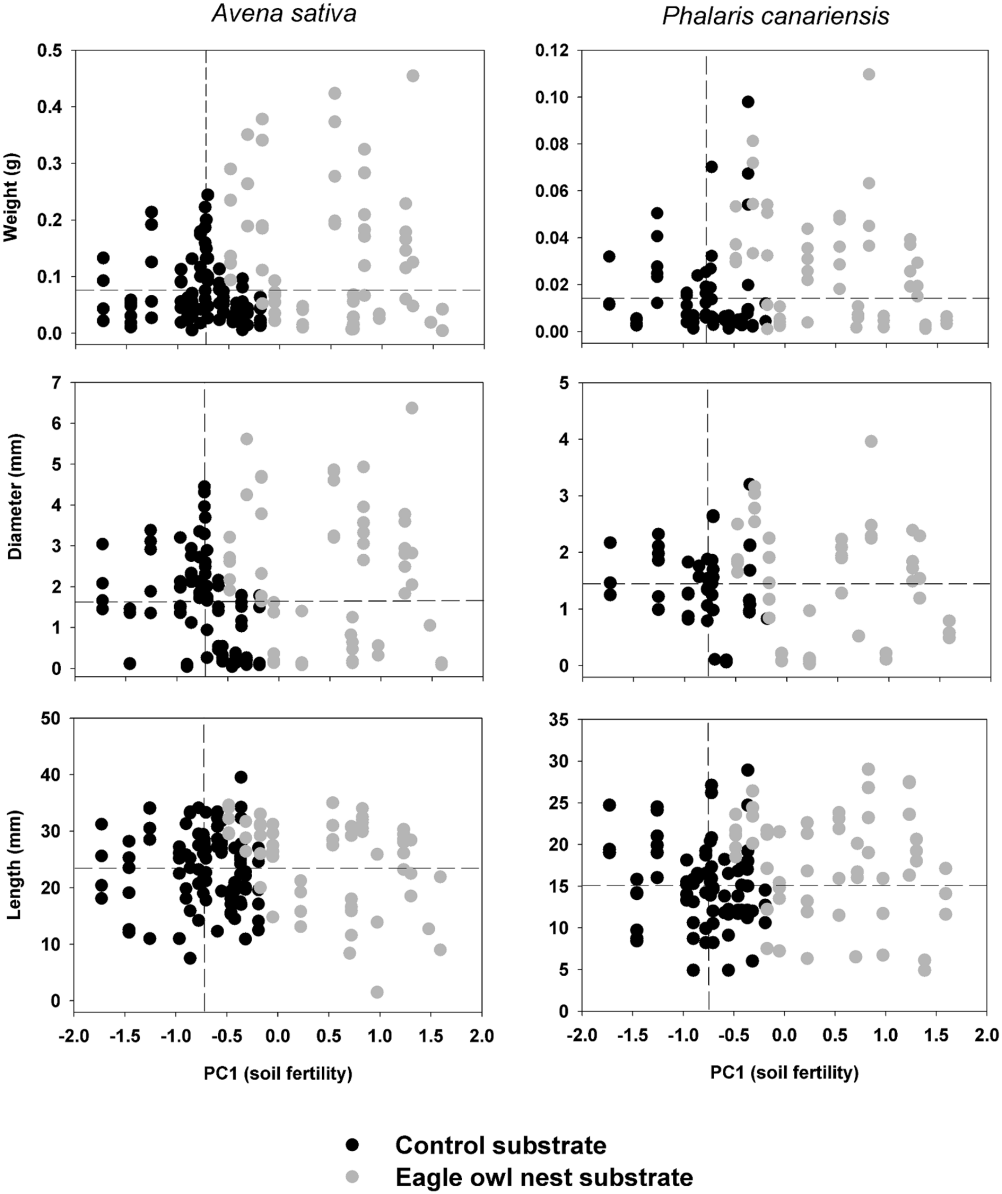
Soil fertility and seedling size. Scatter-plot showing the variation in soil fertility (factor PC1) and different components of seedling size for the two annuals investigated in both control and owl nest top-soils. Discontinuous vertical and horizontal lines represent mean values in control top-soils for PC1 and seedling size, respectively.

Principal components analysis of the correlation matrix of top-soil parameters (across the n = 42 sampling points, all localities combined) revealed the existence of three major independent gradients of variation in top-soil attributes (Table 2). The first component (PC1) clearly corresponds to a fertility gradient, mainly reflecting the coordinated variation of organic matter, phosphorus, potassium, and nitrogen. The positive and negative extremes of the gradient would correspond, respectively, to the most fertile and infertile top-soils. The second component (PC2) mostly corresponds to a top-soil texture gradient running from high clay content on the positive extreme to high sand content on the negative extreme (Table 2). Together, these first two components account for 69% of total between-sample variance. The third component (PC3) accounts for only an additional 16.8% of the variance, and mostly reflects variation in pH (i.e. high pH values on the positive gradient extreme). These three axes thus represent satisfactory simplified descriptors of top-soil characteristics and they were used in the analyses below. Furthermore, a multivariate analysis of variance indicated that there were significant differences between top-soil types in the three gradients overall (F_3, 38_ = 28.40, *P* < 0.0001). However, univariate analyses indicated that these differences were highly significant for PC1 (F_1, 40_ = 77.74, *P* < 0.0001), but non-significant for PC2 and PC3 (*P* > 0.340). Fertility values (i.e. PC1) of owl nest top-soils tended to be much higher than those of control top-soils (Fig 1).

**Table 2:** Principal components analysis of soil texture and chemical composition parameters, conducted on the correlation matrix of soil parameters from sampled owl nests and the corresponding control sites (N = 42).

Soil Parameter	Correlation with principal component (PC)		

	PC1	PC2	PC3
**Sand**	0.306	-0.932	0.193
**Silt**	-0.127	0.859	-0.277
**Clay**	-0.446	0.730	-0.019
**pH**	-0.321	0.219	0.883
**Organic matter**	0.821	0.214	-0.271
**Phosphorous**	0.857	0.252	0.122
**Potassium**	0.578	0.456	0.598
**Nitrogen**	0.917	0.147	-0.051
**Variance explained (%)**	37.45	31.57	16.79

### Seedling growth

**Fig 2:**
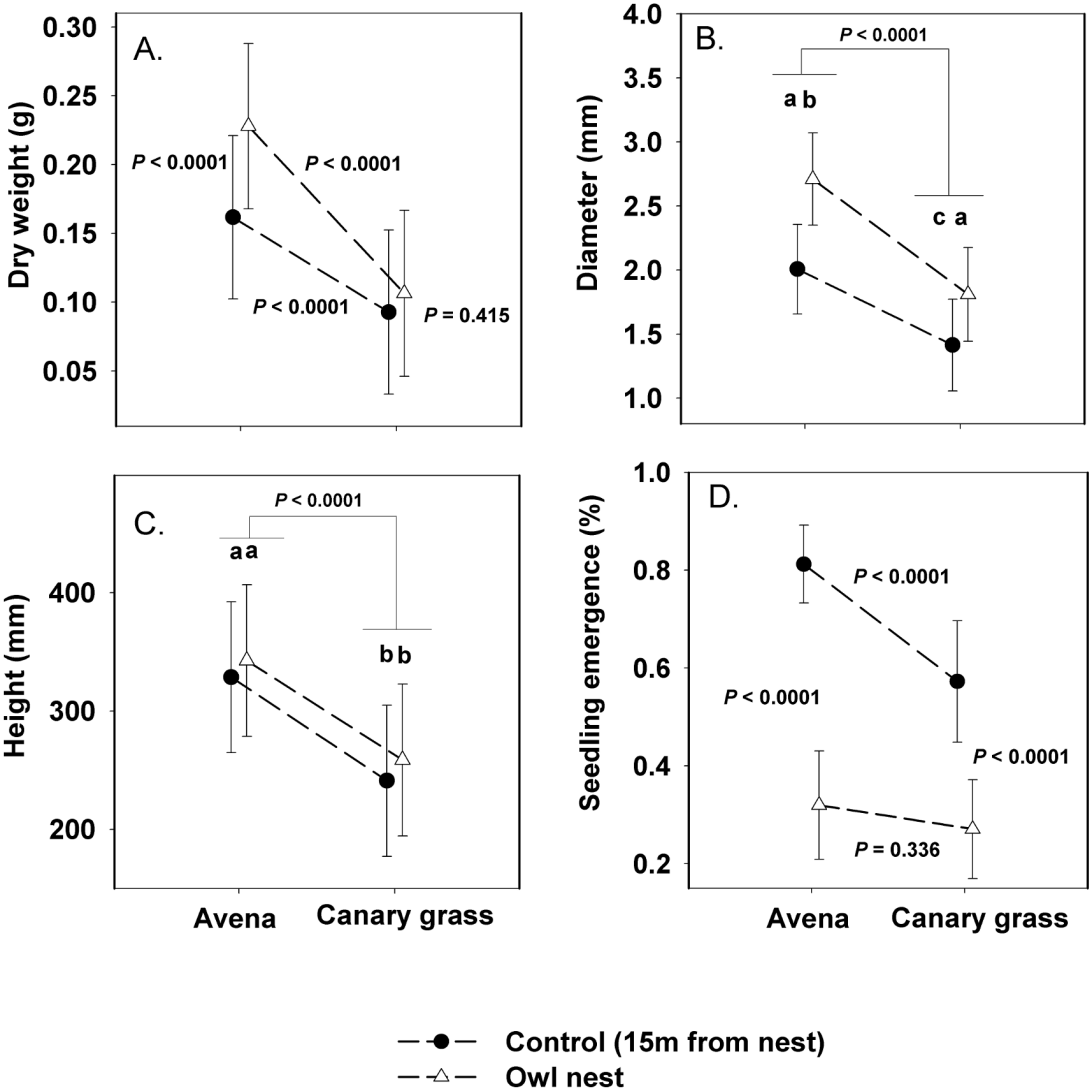
Effect of owl nest on seedling size and emergence. Model-adjusted means (± 1 SE) of different seedling size variables (A, weight; B, diameter; C, length) and the percentage of emergence (D). When the interaction between two variables was significant (A, C), we report the *P*-values of the tests for the four simple main effects involved in the interaction. Different letters denote significant differences.

**Fig 3:**
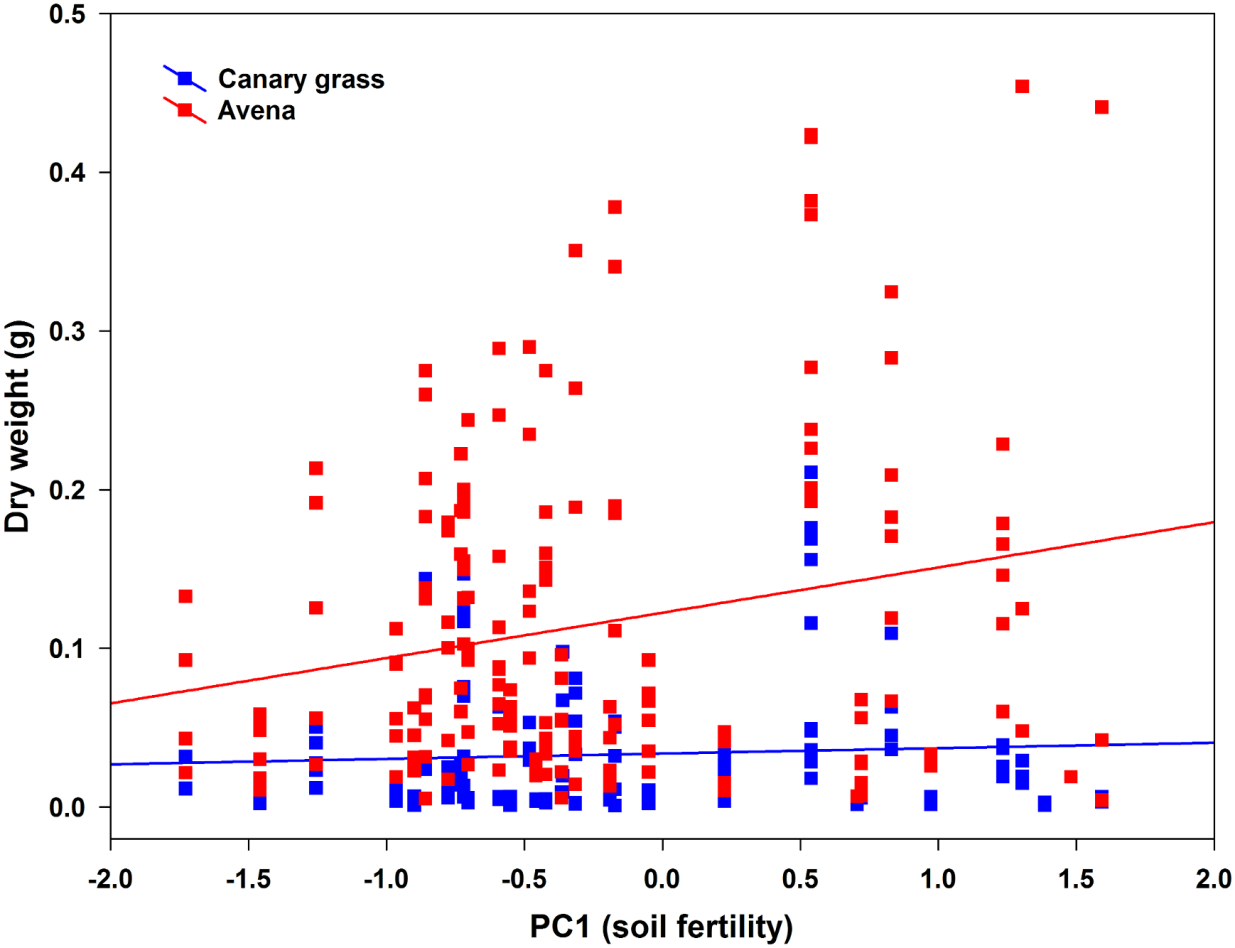
Positive linear relationships between seedling dry weight (g) and soil fertility (PC1) for the Canary grass (r = 0.054, *P* = 0.515, R^2^ = 0.003) and Avena (r = 0.214, *P* = 0.004, R^2^ = 0.046). Note that even though both species show a positive relationship, it only was significant for Avena.

A total of 326 seedlings emerged, 179 and 147 from *A*. *sativa* and *P*. *canariensis* seeds, respectively. Overall, seedling dry weight was on average 1.3 times higher in owl nest top-soils than in control top-soils (*P* < 0.0005; Fig 2A). There was also a marked significant effect of plant species on seedling weight, as *A*. *sativa* seedlings were on average 1.96 times heavier than *P*. *canariensis* seedlings (Fig 2A; Table 3). We found a significant interaction between top-soil and plant species (Table 3), indicating that the effect of one factor was not consistent across the levels of the second factor. Specifically, tests of slices indicated that top-soil type did not have an effect on *P*. *canariensis* seedling weight (F_1, 320_ = 0.67, *P* = 0.415), whereas *A*. *sativa* seedlings emerged from owl nest top-soils were significantly (F_1, 320_ = 18.77, *P* < 0.0001) heavier than those emerged from control top-soils (Fig 2A). As predicted, seedling weight showed a positive significant relationship with PC1 and PC3 (Table 4), indicating that most fertile top-soils (e.g. owl nest top-soils) with a high pH hold heavier seedlings. Not surprisingly, species had a strong main factor effect on seedling weight, but also it showed a non-predicted significant interaction with PC1 (Table 4). Such interaction, however, just indicated that even though seedling weight of both species increased with increases on PC1, the trend was stronger for *A*. *sativa* (Fig 3). Neither PC2 nor PC3 nor any other interaction had significant effects on seedling weight (*P* > 0.081; Table 4).

**Table 3:** Main results of the generalized linear mixed models testing the effects of top-soil and plant species as well as their second-order interactions on different seedling size components (weight, diameter, length) and the percentage of seedling emergence as well.

	Weight			Diameter			Length			Emergence		
	df	*F*	*P*	df	*F*	*P*	df	*F*	*P*	df	*F*	*P*
**Top-soil (S)**	1, 320	12.22	**.0005**	1, 291	25.85	**<.0001**	1, 349	5.05	**0.025**	1, 89	84.49	**<.0001**
**Plant species (P)**	1, 320	72.06	**<.0001**	1, 291	55.76	**<.0001**	1, 349	174.04	**<.0001**	1, 89	14.82	**0.0002**
S*P	1, 320	5.52	**0.019**	1, 291	2.34	0.127	1, 349	0.07	0.793	1, 89	6.63	**0.012**

**Table 4:** Main results of the generalized linear mixed models testing the effects of principal components (based on substrate parameters), annual species, and their interactions on several seedling size components (length, diameter, weight) and the percentage of seedling emergence as well.

	Seedling weight			Seedling diameter			Seedling length			Seedling emergence		

	Parameter estimate	*F*_*1*,*258*_	*P*	Parameter estimate	*F*_*1*,*205*_	*P*	Parameter estimate	*F*_*1*,*262*_	*P*	Parameter estimate	*F*_*1*,*54*_	*P*
**Species (Sp)** †	-0.093	120.44	**<.0001**	-0.951	56.94	**<.0001**	-8.081	101.60	**<.0001**	-0.711	7.92	**0.007**
**PC1**	0.043	18.66	**<.0001**	0.707	35.38	**<.0001**	0.041	0.83	0.362	-1.556	55.55	**<.0001**
**PC2**	0.014	3.06	0.081	0.175	2.01	0.158	-0.392	0.23	0.635	-0.411	0.09	0.762
**PC3**	0.033	4.46	0.036	0.654	13.77	**0.001**	0.909	0.34	0.560	-1.140	19.69	**<.0001**
PC1*Sp †	-0.029	8.21	**0.004**	-0.155	0.97	0.326	1.062	1.22	0.270	0.367	1.47	0.230
PC2*Sp †	-0.002	0.06	0.801	0.029	0.04	0.843	1.442	3.74	0.055	0.703	6.32	**0.015**
PC3*Sp †	-0.022	2.43	0.120	-0.102	0.21	0.649	-0.622	0.23	0.632	-0.031	0.01	0.939

^**†**^The parameter estimates in this row correspond to *P*. *canariensis*, whereas the ones corresponding to *A*. *sativa* were always equal to zero.

Both top-soil and plant species had significant effects as main factors on seedling diameter (Table 3). On average, seedlings emerged from owl nest top-soils were 1.46 times wider that those emerged from control top-soils (Fig 2B). Also, *A*. *sativa* seedlings were 1.35 times wider compared to *P*. *canariensis* (*P* < 0.0001; Fig 2B). There was no significant interaction between top-soil and plant species (Table 3), indicating that the effect of each factor was consistent across the levels of the second factor. As for seedling weight, seedling diameter showed a positive significant relationship with PC1 and PC3 (Table 4), indicating that most fertile top-soils with a high pH hold thicker seedlings. The relationship between seedling diameter and fertility was not linear for *A*. *sativa* or *P*. *canariensis*, and seedling weight tended to decrease for PC1 values greater than 1.0 (Fig 1). Species had a strong effect on diameter effect as main factor and did not show any significant interaction with any principal component (Table 4). Neither PC2 nor PC3 had any significant effect on seedling weight (*P* > 0.081; Table 4).

Seedling length showed a similar pattern to that reported above for diameter (Table 3). On average, seedlings emerged from owl nest top-soils were slightly longer than those emerged from control top-soils (Fig 2C; *P* = 0.025). Also, there was a strong significant effect of plant species as the main factor, as *A*. *sativa* seedlings were 1.34 times longer compared to *P*. *canariensis* seedlings (*P* < 0.0001; Fig 3C). There was no significant interaction between top-soil and plant species (Table 3) indicating, for example, that *A*. *sativa* seedlings were longer than *P*. *canariensis* seedlings in both top-soils. Seedling length did not show any relationship with the principal components describing top-soils (*P* > 0.636; Table 4, Fig 2). There was, however, a marginally significant interaction between species and PC3 suggesting a slightly inconsistent trend between species.

### Seedling emergence

We also evaluated the effect of top-soil on the amount and speed of seedling emergence. Once the block effects were corrected for, our mixed model revealed that both top-soil type and plant species had strong significant effects on the likelihood of seedling emergence (Table 3). In particular, the percentage of emerged seedlings was, on average, 2.40 times higher for seeds sown in control top-soils than for seeds sown in owl nest top-soils (Fig 3D). There was also a marked significant effect of plant species on the percentage of seedling emergence, with 1.42 times more seedlings emerging from *A*. *sativa* seeds compared to *P*. *canariensis* (Fig 3D). Interestingly, however, there was a significant interaction between top-soil and plant species (Table 3), indicating that the effect of one factor was not consistent across the levels of the second factor. For instance, tests of slices indicated that in control top-soils seedling emergence was significantly (F_1, 89_ = 018.46, *P* < 0.0001) higher for *A*. *sativa* than for *P*. *canariensis*, while in owl nest top-soils the difference between species in the percentage of seedling emergence was not significant (F_1, 89_ = 0.94, *P* = 0.336; Fig 3D). In general, seedling emergence started about a week after sowing. The Cox regression analysis indicated that, once corrected for the effect of block, there was a significant difference between species in emergence date (χ^2^ = 11.0, df = 1, *P* = 0.001). Thus, on average, *A*. *sativa* seedlings emerged 1.43 days earlier than *P*. *canariensis* seedlings. Neither top-soil type nor its interaction had an effect on the speed of emergence (*P* > 0.655).

Seedling emergence showed a significant negative relationship with PC1 (Table 4), indicating that most fertile top-soils (e.g. owl nest top-soils) had lower seedling emergence. Similarly, seedling emergence showed a significant negative relationship with PC3 (Table 4), indicating that top-soils with a higher pH had lower seedling emergence. PC2 did not have a significant effect on emergence as main factor, though showed a weak significant interaction with species (Table 4).

### Effect of owl nests on overall seedling biomass

Finally, because owl nests showed conflicting effects on different aspects of seedling performance (i.e. increased seedling growth but decreased seedling emergence), we evaluated the overall owl nest effect on seedling biomass on a per-pot basis. Overall per-pot seedling dry weight was slightly higher in owl nest top-soils (0.410±0123) as compared to control top-soils (0.359±0.131), though these differences were not significant (F_1,59_ = 0.72, *P* = 0.400). Consistently with above findings, overall per-pot seedling dry weight was 2.7-fold higher for *A*. *sativa* (0.564±0.122) than for *P*. *canariensis* (0.205±0.125; F_1,59_ = 36.08, *P* <0.0001), with these differences being consistent between both top-soil types (i.e. the interaction between species and top-soil type was non-significant; *P* = 0.400).

## Discussion

This study illustrates for the first time how widely distributed inland predators can enrich the top-soil in the immediate surroundings of their nesting places and how this, in turn, enhances plant growth. Our experimental approach allowed us to establish causality between top-soil enrichment and seedling growth enhancement given that, by using germination chambers, we controlled for potential confounding factors such as differences in environmental variables other than top-soil between nests and control samples (e.g. water and light availability; [35]). The results observed for our two target annual grasses are likely applicable to other annuals and possibly also to some perennials [14]. Though we have not measured vegetation cover and species composition in the field, we have also observed higher cover of both perennials and shrubs (e.g. *Asparagus* spp.) in the surroundings of eagle owl nests as compared with similar nearby microsites (Authors *personal observation*). Therefore, our study supports that pervasive inland predators (birds, mammals) exert similar ecological effects at inland habitats as those documented for colonial seabirds in coastal habitats [7]. To fully understand the lack of a significant owl nest effect on overall seedling biomass, and thus to disentangle the role of presumed critical factors (e.g. spatial variation in seed limitation; [36]), a combination of further well-replicated experiments and field measurements of vegetation will be needed.

### Top-soil enrichment in the nest vicinity

The differences between the top-soil nutrient concentration from eagle owl nests and control sites (Table 1) were likely the result of faeces deposition, as well as decomposition of pellets, prey remains and feathers. This suggests that the activities of non-colonial birds at nesting places have the potential to locally increase top-soil nutrients and, consequently, have an important effect on the surrounding vegetation [6, 8, 37, 38]. A similar pattern has been observed for other predators [4, 13, 39, 40]. For example, arctic foxes *Alopex lagopus* L. in the subarctic mountain tundra of Sweden produce a nutrient enrichment around their dens by depositing faeces, urine and carcasses [16, 41]

Our results suggest that ground nesting inland birds may represent an overlooked yet widespread mechanism generating patchiness in nutrient distribution across landscapes in many inland habitats worldwide. Therefore, further studies in other inland predators are clearly needed to evaluate the pervasiveness of such a subtle, but ecologically relevant, mechanism as well as to identify its likely ecological outcomes at the population, community, and whole food-web levels [2].

### Seedling growth enhancement

Our experimental results indicate that nutrient enrichment by nesting owls enhanced seedling growth in both of the annual species investigated. Also, as expected, the effect of top-soil enrichment on seedling growth was most noticeable for the larger and more nutrient demanding *A*. *sativa* seedlings [19–21]. These results are in accordance with previous studies of colonial seabirds which demonstrate that primary productivity is higher in areas occupied by seabird colonies as compared with areas without seabirds [6, 42].

Our study also showed that the percentage of seedling emergence was greater in control sites than in nests which, by increasing intraspecific competition, could have affected seedling growth. There are at least two possible non-mutually exclusive causes to explain the lower number of seedlings emerging from owl nest top-soils. First, an excessive amount of nutrients (N, P) in samples from some eagle owl nests (see Fig 2) could have inhibited seed germination and/or seedling emergence [43–45]. Second, prey remains (bones, skin, etc.) may have covered seeds and, as a result, physically impeded seedling emergence in an analogous manner to that by which litter often reduces the emergence and recruitment of both annual and woody species [46]. Dissecting the relative importance of both mechanisms for contrasting plant species and ecological contexts is certainly a relevant pending task.

Owls and several other local ground nesting birds (partridges, larks, wheatears, nightjars, etc) are likely to markedly increase the spatial heterogeneity in nutrients (P, N, organic matter) at a landscape scale in inland habitats. Spatial heterogeneity results in microhabitat diversity and influences the spatial patterning of different species in a community [9, 47]. Similar results to those observed for the eagle owl can be expected in other ground nesting solitary birds, including wader birds, tetraonids, and bustards. However, the effect is likely to be stronger on nidiculous than nidifugous species, because the former spend more time at the nest (both parents and chicks) and thus have a higher potential to enrich the soil of the nest surroundings. Moreover, the soil enrichment generated by ground nesting birds is most likely to benefit plant species with higher nutrient requirements than less demanding species [48]. Finally, from an animal perspective, nutrient patchiness enhances the growth and establishment of plants around the nest, which may provide greater protection against predators and/or amelioration of severe climatic factors. Thus, the interaction between ground nesting inland predators and vegetation might also generate a number of complex direct and indirect effects on both the plant and the animal side that certainly need to be investigated in more detail under field conditions [2].

In conclusion, our study revealed that owls and other widespread inland predators can exert subtle effects enhancing plant growth and probably also primary productivity through mechanisms (i.e. nutrient enrichment) other than the well-recognized control of primary consumers [7]. Further studies in other inland predators and under contrasting field conditions would help to evaluate whether the reported effects on seedling growth are consistent throughout different plant ontogenic stages (sapling, adults) as well as the pervasiveness of such neglected but ecologically relevant mechanism. The combined effect of nutrient enrichment by non-colonial birds coupled with direct and indirect seed dispersal [4–5] is likely to have a strong effect on community assemblage and ecosystem functioning and represents an overlooked avenue of research that certainly deserves further investigation.
